# Financial performance of rural banks in Indonesia: A two-stage DEA approach

**DOI:** 10.1016/j.heliyon.2020.e04390

**Published:** 2020-07-17

**Authors:** Wasiaturrahma, Raditya Sukmana, Shochrul Rohmatul Ajija, Sri Cahyaning Umi Salama, Ahmad Hudaifah

**Affiliations:** a Department of Economics, Universitas Airlangga, Indonesia; b Department of Shariah Economics, Universitas Airlangga, Indonesia; c Universitas Muhammadiyah Malang, Indonesia; d Department of Islamic Economics, Universitas Internasional Semen Indonesia, Indonesia

**Keywords:** Efficiency, Conventional and Islamic rural bank, Data envelopment analysis, Tobit, Banking, Corporate finance, Econometrics, Financial economics, Microeconomics

## Abstract

This study aims to analyze the efficiency performance of conventional and Islamic rural banks in Indonesia, specifically, *Bank Perkreditan Rakyat* (BPR) and *Bank Pembiayaan Rakyat Syariah* (BPRS). Using a DEA approach, the results indicate that both BPR and BPRS are still inefficient in terms of the intermediation role but are efficient in production. Furthermore, the Tobit estimation show that these two efficiency results are positively affected by location and the capital adequacy ratio (CAR). These rural banks operating in cities tend to have a higher level of efficiency than otherwise. Moreover, the higher the capital, the more efficient both Islamic and conventional rural banks in terms of production and intermediation.

## 1 Introduction

Microfinance institutions (MFIs) are alternative financial providers for communities that are not covered by the banking sector. Most MFIs operate in developing countries (Forcella and Hudon, 2016), such as Sri Lanka (Alawattage et al., 2018), Senegal (Scanlon et al., 2019), India (Baland et al., 2019), Indonesia (Adnan and Ajija, 2015), and other developing countries. In these countries, the existence of microfinance institutions is very important as it affects household loans from information sources in the village economy and provides access to new business opportunities (Islam et al., 2015). In Indonesia therefore, the potential demand in microfinance includes the self-employed and those with no paid workers (Nashihin, 2014).

There are some classifications of MFIs in Indonesia. According to the principles, there are two categories of MFIs, i.e. Islamic and conventional MFIs. The difference between the two lies in the operations of Islamic MFIs that are based on sharia, such as no interest and clear contract (Aburime, 2008). Islamic MFIs in Indonesia usually promote equity and trading products (Anwar, 2016), while, interest based is utilizied by the counterpart. Moreover, based on their legal entity, according to Law No. 1 of 2013, MFIs in Indonesia can be in the form of bank supervised by Indonesia Financial Services Authority (OJK) and non bank controlled by Ministry of Cooperatives and Small and Medium Enterprises (SMEs).

Rural banks, known as *Bank Perkreditan Rakyat* (BPR) are one type of MFIs developed in Indonesia. With the issuance of Law No. 7 of 1992 as amended to Act No. 10 of 1998 concerning Banking, these institutions officially became operational. In the Act, it is explained that these are banks that carry out business activities in a conventional manner based on sharia principles (termed as *Bank Pembiayaan Rakyat Syariah/*BPRS) that do not provide payment traffic services in their activities. Thus, Rural Banks have several differences with Commercial Banks. First, they have capital requirements that are much smaller than Commercial Banks. Second, their target is to serve the credit needs of farmers, fishermen, small traders, employees, retirees, and other layers of society that have not been reached by their counterparts to prevent them from being trapped by moneylenders (Iswandari and Anan, 2015). Therefore, the services provided by rural banks are incomplete compared to those offered by commercial banks such as insurance, credit cards, demand deposits, and foreign exchange. Accordingly, it is no doubt that rural banks both Islamic and conventional are also part of MFIs in Indonesia.

Those institutions have important roles in driving the development of economy in Indonesia. Although credit to SMEs is still dominated by commercial banks, the contribution of BPR and BPRS continues to increase from 4.79 percent in 2013 to 7.5 percent in 2017. The total SMEs loans disbursed by BPRs and BPRS continues to increase, specifically, from 30.65 Trillion Rupiah in 2013 to 46.68 Trillion Rupiah in 2017. Furthermore, they also have specific business activities that serve SMEs and aim to help build the local economy (Wahyuni et al., 2014).

According to Indonesia Financial Services Authority (OJK), there were 167 Sharia Rural Banks and 1,619 Conventional Rural Banks in 2017, and this distribution is still dominated in the western region. The ten provinces with the highest number of BPR are in East Java, West Java, Central Java, Bali, West Sumatra, Banten, North Sumatra, D.I Yogyakarta, and Riau. While those with the lowest number are in Central Sulawesi, Bengkulu, Central Kalimantan, Nangroe Aceh Darussalam, West Papua, Gorontalo, Bangka Belitung, North Maluku, Maluku, and West Sulawesi.

The tremendous development of BPR and BPRS needs to be balanced with excellent financial performance. This is important due to their small market share which is based on micro businesses with high default risk (Firmansyah, 2014). According to the Indonesia Deposite Insurance Aggency or *Lembaga Penjamin Simpanan/LPS* (2019), from January 2006 to August 2016, 72 units or 4.4 percent BPRs and two units or 1.2 percent BPRSs were liquidated. Moreover, there were 24 BPR and BPRS in the liquidation process from September 2016 to July 2019 due to their inability to compete in the banking industry and the numerous acts of fraud committed by the management or owner of that micro bank which lead to criminal cases (Rustiarini et al., 2016). Furthermore, similar to Islamic commercial banks that conduct their business based on Sharia principles, BPRS and BPRs are not expected to repeat the same mistakes. Nevertheless, during that period, two BPRS were liquidated and four were in the process of liquidation.

Understanding the financial performance of all BPRs and BPRS in Indonesia is absolutely essential. This is because, some previous studies separately analyzed separately the BPR and BPRS, such as Fauzi (2014), Muhari and Hosen (2014), Trinugroho, et al. (2018) Paramita (2008) Hartono et al. (2008), and Septianto and Widiharih (2010). Research which comprehensively analyzes financial performance has indeed been carried out for Islamic and conventional commercial banks such as those conducted by Anwar (2016).

This research, therefore, contributes to evaluate the efficiency of both conventional and Islamic rural banks in the last five years especially after OJK officially started operation, i.e. 2013 to 2017. In the second part, this paper presents several literature reviews about efficiency analysis of financial institutions. Section 3 describes research data and methods for evaluating the rural banks’ efficiency while Section 4 provides the findings and discussion. Finally, the last section summarizes the findings and their implications.

## 2 Literature review

### 2.1 Basic principles of BPR and BPRS

BPR and BPRS have fundamental differences in terms of the principles utilized. Similar to other Sharia-based financial institutions, BPRS runs its business processes based on Islamic values with interest free rates (Iqbal, 1997). This means that every financial transaction in an SRB may not have an element of usury *(riba)* or in this case interest on the loan.

The imposition of this usury is forbidden by Allah in accordance with Surah Al-Baqarah Ch.1: verse 275, therefore, it is a threat a threat for Muslims not to apply it in all their transactions. Accodring to Ziyadah, the term usury means additional, while Saeed (1996) defined it as the process of growth. Terminologically, usury is interpreted as an additional return from vanity of basic assets (Chaudhry, 1999:4). Fatwa National Sharia Board number 1 of 2004 stated that the practice of interest transactions which occurs at this time has fulfilled the criteria of the Prophet mas'ah, therefore the haram law carried out by various financial institutions (Majelis Ulama Indonesia, 2004). Furthermore, the SRB may not provide additional loans received or channeled to customers. BPRS provides or receives benevolent loans in the form of Qardh contracts, with no additional interest on the loans.

In contrast to the BPRS, BPRs use debt contracts with customers, when they place their funds to obtain interest on the money saved. Instead, funds are lent to customers, with interest charges on the loans disbursed (Yuwana et al., 2012). This business activity is prohibited in Islam because it is considered an element of injustice where creditors provide loans on the conditions of return accompanied by payment of interest which is fixed and determined at the beginning of the transaction. In running a business, the borrower does not always obtain profits (A. Ahmad, Rehman and Humayoun, 2011).

BPRS implements several contracts in running its business in order to avoid usury. In terms of fund raising, it tends to utilize wadiah yad adh-dhamanah (deposit) and mudharabah agreement. While in the wadiah yad adh-dhamanah contract, the funds are used for business with the availability of funds when neeed by the owner (Ajija et al., 2018). Mudharabah contract, provides profit sharing to customers with the principle of revenue or profit/loss sharing. Generally, all BPRS in Indonesia use revenue sharing according to the ratio agreed at the beginning of the contract. Due to profit/loss sharing, there is a possibility that customers also bear losses and of course this affects decision to save funds in Islamic financial institutions. Therefore, the profit sharing obtained by the customer is dependent on how much income or profit the SRB acquires in that month (Beck et al., 2010).

In terms of channeling funds, BPRS uses contracts based on trading (*murabahah*), equity (*Mudharabah/Musharakah*), or leasing (*ijarah*) (Amelia and Fauziah, 2017) due to most of its distribution in the form of trading. Therefore, BPRS tends to benefit from buying and selling transactions, revenue sharing from equity-based transactions, and fees (*ujroh*) from leasing transactions. In addition to using a Sharia-compliant agreement, the distribution of funds to the community need to pay attention to Islamic principles of morality which are regulated in the fiqh al-muamalah. Therefore, IFI is prohibited from investing in immoral or illicit businesses such as alcohol, gambling, pork, pornography, hoax or gossip news media, and others (Ika and Abdullah, 2011).

### 2.2 Banking efficiency

Efficiency is used to measure the value of output produced from a number of inputs used, and this involves measuring company performance (Al-Darrab, 2000). When the output of a company is equal to or greater than its input, it is declared efficient. Two approaches are used in measuring efficiency in microfinance institutions, namely, the intermediation and production approaches. The production approach assumes that the company as a producer generates savings and loan accounts, while the intermediation approach assumes that the company's activities transform money borrowed from surplus funds to the deficit (Ahmed, 2002; Khan, Amin, Khokhar, ul Hassan and Ahmad, 2018).

Efficiency is improved in various ways such as increasing the consolidation of MFIs and profitability (Hartarska et al., 2013). Contrarily, the small size of loans reduces the level of efficiency (Bos and Millone, 2015). The profit factor or the amount of margin used also influences the performance, including Sharia Microfinance Institutions (Amran et al., 2014; Hudon and Périlleux, 2014).

There are strategies to maintain the existence of microfinance institutions in the midst of the various banks currently in micro-communities, one of which is operating, efficiently (Nashihin and Harahap, 2014). However, there is no doubt the strategy for microfinance industries in each country is different, as is the case in MENA which requires a strategy to develop technology to further embrace the poor and financial sustainability (Bassem, 2014).

So far, the technical efficiency of financial institutions and Islamic banking is superior compared to conventional but the average cost efficiency is much lower due to cost inefficiencies and allocation errors (Rosman et al., 2014; Zuhroh et al., 2015). This is as a result of the diversification of the income and ownership status of financial institutions which has proven to have an influence on their efficiency. The status of state ownership does not affect the level of efficiency in microfinance institutions both in developing and developed countries, but private ownership in developing countries tends to be efficient especially after a crisis occurs (Doan et al., 2017).

Currently, microfinance institutions are faced with two conditions, namely, maintaining the ideology to improve the welfare of the poor and pursuing profits (Kaur, 2016). There is a trade-off between outreach to poor people and cost efficiency (Abate et al., 2014). Many MFIs are more financially efficient than socially (Abate et al., 2014).

Research on efficiency is mostly carried out in developing countries because of the existence of the most active MFIs. The GCC region (Alqahtani et al., 2017), Middle Eastern and Asian countries (Rosman et al., 2014) (Rosman et al., 2014), Sri Lanka (Wijesiri et al., 2015), and Indonesia (Farida et al., 2018) most often use Data Envelopment Analysis (DEA) as a tool to measure this factor. Other methods besides DEA include Development Economic Analysis (Hudon and Périlleux, 2014; Nashihin and Harahap, 2014), Stochastic Frontier Analysis (SFA) (Nurboja and Košak, 2017), OLS and Tobit (Bitar et al., 2017), and profit models (Berge et al., 2016).

DEA is commonly used because it is different in terms of measurement. First, the measurement of efficiency is technical therefore it only takes into account the absolute value of a variable. The resulting values are relative, thus, they are only applicable to the tested unit (S. Ahmad, Rahim and Rahman, 2012). Previous studies may lack adequate methods or further research, hence, this study bridges the gap by using DEA and Tobit.

The variables used in this study are different from previous research, such as, only employee salary costs, operating costs and other expenses (Bibi et al., 2017), company size, gross domestic product, capital, liquidity, profits, and inflation (Fernandes et al., 2018), microfinance equities and daily quote prices (Brière and Szafarz, 2017). Compared with previous research which only used one approach, this study will use two approaches simultaneously, namely, the production and intermediation. In the production approach, the output variables used are interest/margin receipts/profit sharing from loans channeled and other revenues, while the input variables are interest/margin/profit sharing, expenses for productive assets, administrative and general expenses as well asother expenses and non-operating expenses. The intermediation approach uses a channeled loan output, while the input variable consists of capital, liabilities that can be paid immediately, savings, deposits, bank loans, and total assets. Furthermore, the objects of this research are BPR and BPRS which have rarely been used in other studies.

## 3 Data and research methods

### 3.1 Data

The data used in this study are secondary and obtained from the financial statements of BPR and BPRS for the last five years starting from 2013 to 2017 which have been available on the pages of the Financial Services Authority. Rural banks and Sharia Rural Banks with incomplete financial statements for the last five years are not used as samples. Based on data compiled by OJK, up to 2017 there were 1,619 BPR and 167 BPRS. After categorization, 1,271 BPR and 113 BPRS were used as research samples.

Sidoarjo and Badung Regency are the districts with the highest number of BPR samples. The total number in Sidoarjo is 55 units, but only 50 units have complete financial reports. In Badung Regency, Bali, the total number was 51 units but only 49 fit sample criteria. Supposing the districts and cities in Java Island have many rural banks that meet the sample, districts and cities in Java Island have many rural banks that meet the sample criteria, while there are few in external regions, especially in Kalimantan, Maluku, and other central and eastern parts of Indonesia. Most have only one or two BPRs in existence for over five years.

Most regions in Indonesia only have one BPRS sample used in this study. This is because the number of BPRS in Indonesia is still not as much as BPR. Regions that have 2 BPRS samples, namely Mataram City, Gresik Regency, Bandung City, Bandar Lampung City, Makassar City, Kediri Regency, Tangerang City, Yogyakarta City, and Agam Regency. Regions that have 3 BPRS samples, namely Semarang City, Pasuruan City, Bekasi City, Bantul Regency, Kampar Regency, Banyumas Regency, Solo City, Bekasi Regency, Depok City, Bogor Regency, Cilacap Regency, and Sidoarjo City. While the regions that have more than three BPRS as samples are Bandung and Sleman regencies with a total sample of 5 BPRS per region and Serang Regency with 4 samples (Anwar, 2016; Ibrahim, 2019; Rustiarini et al., 2016).

### 3.2 Research methods

Measurement of Conventional and Sharia rural bank efficiency is carried out using a non-parametric Data Envelopment Analysis (DEA). The focus of this measurement is on the contribution of technical change in the scale of Total Factor Productivity (TFP). Due to the analysis condition not being input oriented, output-orientation is used here and in dynamic measurements. Furthermore, the DEA CCR output models (Charnes, Cooper, and Rhodes) and BBC (Banker, Charnes, and Cooper) with Variable Return to Scale (VRS) were used to measure the efficiency of MFIs.

According to Holod and Lewis (2011), there are many studies measuring the efficiency in the banking industry especially after the works counducted by Green (1967) and Benston (1965). There are two approaches in selecting input and output variables, namely production and intermediation (Syamni and Abd Majid, 2016). In the production approach, the MFI input includes all the operational costs used to produce various types of assets while the output is in the form of loans and deposits or third-party funds. When this is the case, then the input only covers operational costs and not deposits or interest paid for deposits. In the intermediation approach, the MFI is seen from its role as a liaison between savers/depositors and investors. The output is measured in money value, while total costs include operating costs and interest expenses. This study will measure the level of efficiency of conventional and sharia BPR/BPRS with production and intermediation approaches. Input and output variables called Decision Making Unit (DMU) are used as represented in the following Table 1:

**Table 1:** Efficiency measurement input and output.

Approach	Input	Output
Production	1. Interest/margin/profit sharing from third-party fund 2. Expenses for Allowance for Earning Assets 3. Administrative and General Expenses 4. Non-operational expenses 5. Other expenses	1. Receipt of interest/margin/profit sharing from loans disbursed 2. Other revenue
Intermediation	1. Capital 2. Savings 3. Time Deposits 4. Bank Loans	Loans/financings disbursed

In the production approach, the determination of input and output variables is based on the Cobb Douglas production function. Furthermore, the output variable is the income received by the MFI while the input includes all possible costs that arise from capital and labor (Vujcic and Jemric, 2001).

Specifically, there are fundamental differences in concepts in the input and output variables of BPR and BPRS. Revenues earned are in the form of interest income from funds loaned to customers or placed in banks, with penalties for late payments. While income from BPRS is obtained from Sharia financing and loan transactions in the form of buying and selling/*murabahah*, fees for services/*ujroh*, and profit sharing on mudharabah and musyarakah contracts. Penalties due to late payment of installments by customers are not included in the income of the BPRS but are in social funds, therefore, they are not included as output in this study. While in the input aspect, the costs of BPR are in the form of interest paid to customers' funds or other banks that save their money in the form of savings or time deposits. The costs are in the form of profit sharing from customers' deposits, using mudharabah contracts and bonuses on wadiah (Anwar, 2016; Devi and Firmansyah, 2018; Ibrahim, 2019; Rustiarini et al., 2016).

In the intermediation approach, input and output variables are determined to measure the efficiency of MFIs in collecting and channeling funds. Therefore, the output used channeled loans because the main activities of conventional and sharia BPR/BPRS involve funds for customers success rate to be measured through total revenues including interest income/margin/profit sharing (Khan et al., 2017; Ochola, 2016; Sebhatu et al., 2013). The input variable includes all sources used for channeling capital and debt funds. However, there is no debt in BPRS except *qard* or loan virtues, therefore, to generate income, the BPRS conducts buying and selling transactions and business cooperation in the form of *mudharabah* and *musyarakah* with customers (Devi and Firmansyah, 2018).

After complete input and output data on BPR and BPRS are obtained, then we calculate efficiency scores for BPR and BPRS. We do not interfere with BPR data with BPRS. This means that the efficiency score obtained by BPR is relative to other BPRs and does not involve BPRS. We also do the same thing when calculating BPRS efficiency scores. The aim is so that the assessment of the efficiency of BPR is not biased with BPRS considering that institutionally BPR has existed far earlier than the BPRS so that it will be unfair if the input and output components are equalized (Anwar, 2016).

This research is different from Syamni and Majid (2016) in which the efficiency of intermediation of microfinance institutions was measured by taking cooperative case studies in North Aceh, Indonesia. In their study, total business volume or revenue was used as the output variable, but instead, this study uses outstanding funds because the purpose of the intermediation approach is to measure how efficient a financial institution is. This is made more specific to the intermediation aspect and does not involve the success of financial institutions in obtaining actual income, which is the focus of the production aspect.

The financial conditions of the two institutions analyzed by the DEA method from 2013 to 2017 can be seen in Table 2. From the aspect of intermediation, the overall performance of BPRS appears to be better than BPR. This is evident in the average credit channeled by BPRS which tends to be higher than BPR because public funds in the form of savings and time deposits are also much greater. In addition, BPRS capital appears to be larger than BPR and from the production aspect, it can be seen that BPRS revenues and costs are also greater. This is certainly reasonable considering that the funds channeled by BPRS to the community are much higher.

**Table 2:** Descriptive statistics of input and output of DEA model (in Million IDR).

Variables	Mean	STD	Min	Max
**BPR**				
Loan disbursed	12,919.01	10,752.25	5,316.03	20,522.00
Capital	2,050.00	70.71	2,000.00	2,100.00
Savings	3,491.05	2,994.04	1,373.95	5,608.16
Time Deposits	5,996.58	3,419.46	3,578.65	8,414.50
Bank Loan	2,292.01	3,135.89	74.59	4,509.42
Receipt of interest from loans disbursed	3,539.00	3,318.79	1,192.26	5,885.74
Other revenue	240.71	164.44	124.44	356.99
Interest from third-party fund	705.75	585.00	292.10	1,119.41
Expenses for Allowance for Earning Assets	185.42	262.22	0.00	370.83
Administrative and General Expenses	1,519.00	1,141.69	711.70	2,326.29
Other expenses	37.60	44.68	6.01	69.20
Non-operational expenses	46.68	55.95	7.12	86.25
**BPRS**				
Financing disbursed	41,155.10	81,872.79	1,007.60	794,740.46
Capital	6,239.37	10,730.34	500.00	96,000.00
Savings	13,333.92	23,336.14	17.54	203,807.20
Time Deposits	21,374.63	47,553.15	105.00	359,322.75
Liabilities to other banks	698.81	3,644.60	0.00	41,166.03
Receipt of margin/profit sharing from financing disbursed	31,470.90	44,151.58	251.01	62,690.78
Other revenue	2,606.61	3,608.45	55.05	5,158.17
Margin/profit sharing from third-party fund	12,229.25	17,234.53	42.60	24,415.90
Expenses for Allowance for Earning Assets	635.99	859.85	27.99	1,244.00
Administrative and General Expenses	13,931.01	19,146.86	392.13	27,469.89
Other expenses	274.67	335.06	37.75	511.59
Non-operational expenses	53.01	62.38	8.90	97.12

Furthermore, the data summarized in Table 2 are processed using the DEA model to obtain a technical efficiency score with an output-oriented variable return to scale approach. Generally, the DEA models for this research are as follows:

(1) MinΦs.t.

(2) $$\sum_{j}\lambda_{j}x_{jm}\leq \text{Φ}x_{j_{0}m}\;\;;m=1,2,\ldots M$$

(3) $$\sum_{j}\lambda_{j}x_{jn}\geq \text{Φ}x_{j_{0}n}\;\;;n=1,2,\ldots N$$

(4) $$\lambda_{i}\geq 0\;;j=1,2,\ldots .J$$

Where: Φ is DEA efficiency inverse, $x_{jm}$ is the input *m* from DMU *j*, $j_{0}$ is DMU, $y_{jn}$ is the output *n* for DMU *j*, and $\lambda_{j}$ is the variable to be calculated from the data.

After determining the efficiency score through both production and intermediation approaches, the factors that influence the score will be estimated using panel Tobit. The bank's financial performance is influenced by business scale, CAR, ownership structure, market share, market concentration, and NPL. Banks that have a high business scale tend to reduce production costs because they have reached a high economic scale (Bikker and Hu, 2002; Guillén et al., 2014; Pasiouras and Kosmidou, 2007; Short, 1979; Smirlock, 2006). A good bank is one that can maintain a high level of CAR because it can reduce its bankruptcy rate (Liu and Wilson, 2010; Pasiouras and Kosmidou, 2007).

In general, a private-owned bank will be more powerful than the state-owned bank (Nouaili et al., 2015). This is due to state-owned banks often bearing more risky loans, namely customers with a high risk of default and not having good asset quality (Cornett et al., 2009). A bank with a weak market share usually tends to have poor performance (Liu and Wilson, 2010). In addition, a high NPL usually has a negative effect on a bank's performance (Georgievska et al., 2011). The determinant of efficiency scores in this study is also seen from the possibility of internal and external factors. The location also greatly determines the success of MFI efficiency (Ferdousi, 2013). Furthermore, the age of MFIs and the number of offices is considered to have a positive effect on their efficiency (Akram et al., 2016). In summary, the variables used in the panel Tobit are provided in Table 3.

**Table 3:** Variables of Tobit model.

Variables	Description of Variables
TE-Prod	Technical Efficiency of the production approach
TE-Inter	Technical Efficiency of the intermediation approach
Car	The capitalization rate is measured by the ratio of capital to total assets (capital adequacy ratio/CAR)
NPL/NPF∗	Non-performing loan/financing in percentage
Owner	Ownership structure (1 represents government property, and 0 is others)
Loc	Location of office (1 indicates city, and 0 is others)
Scale	Total asset in natural logarithm

∗ Note: NPL is non performing loan di BPR, and NPF is non perfroming financing in BPRS.

## 4 Findings and discussions

In Indonesia, BPR emerged in 1977 since PT. Bank Rakyat Indonesia (BRI) began to develop village barns, market, village, employee and other similar banks. By De Yure, BPR was first recognized in the De facto on October 27, 1988, as part of the Financial, Monetary and Banking Policy Package. Furthermore, the institution is basically a new name for several financial institutions built by BRI namely Bank Desa, Lumbung Desa, Bank Pasar, Bank Pegawai Lumbung Pilih Nagari (LPN), Lembaga Perkreditan Desa (LPD), Badan Kredit Desa (BKD), Badan Kredit Kecamatan (BKK), Kredit Usaha Rakyat Kecil (KURK), Lembaga perkreditan Kecamatan (LPK), Bank Karya Desa (BKPD) and other similar institutions. Furthermore, since the issuance of Law No. 7 of 1992 concerning Principal Banking, the financial institution has clearer legal status through the Minister of Finance.

In a further development, BPRs were not only managed conventionally using the interest system but also began to be managed using Islamic financial principles, hereinafter referred to as BPRS. In Indonesia, the BPRS that first operated were PT. BPR Dana Mardhatillah, PT. BPR Berkah Amal Sejahtera, and PT. BPR Amanah Rabbaniyah in 1991, located in Bandung, West Java. Thus, BPR existed long before BPRS.

The inefficiency in carrying out the intermediation role shows that the two microfinance institutions still cannot optimally channel their funds to the community. This means that many of the collected funds are not financially channeled to customers. Considering that the market shares of the two institutions are micro, small and medium enterprises tend to have high business risks. In addition, BPR and BPRS managers have calculated that with their current conditions of intermediation, they have succeeded efficiently from the production aspect, meaning they succeeded in achieving optimal revenue at an efficient cost.

**Figure:**
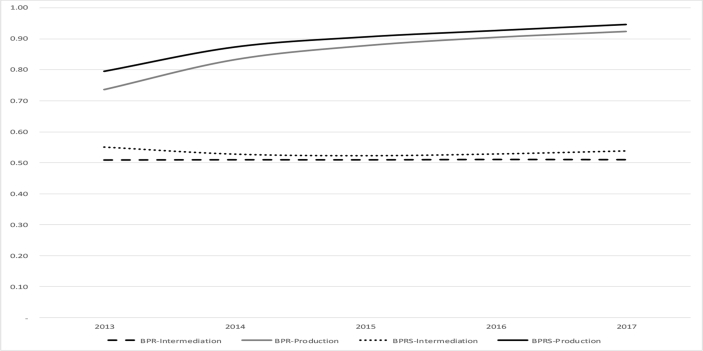
Picture 1: The Average of Technical Efficiency of BPR and BPRS.

From these calculations, the efficiency level of BPR and BPRS in the observation period is higher than the intermediation efficiency. In 2013 to 2017, with a trend that was often stagnant, the average efficiency of BPR intermediation was 0.51 while that of BPRS was 0.53. Meanwhile, as the trend increases yearly, the average production efficiency of BPR is 0.86 and 0.89 for BPRS. When the efficiency score is between 0.40 to 0.60, the company is still said to be inefficient, while if it is between 0.80 to 1.00, then the company is said to be efficient. Based on the grouping, it can be concluded that by using the intermediation approach, both BPR and BPRS are still inefficient. Meanwhile, they achieved efficient conditions in terms of the production approach.

More specifically, there are several BPRs and BPRS that have the potential to be efficient in carrying out their intermediary roles (see Table 4). However, these two cannot be compared because in this study, BPR and BPRS are separated in efficiency analysis. Thus, the number of Decision Making Units (DMUs) analyzed is not the same and the efficiency score of BPR applies to it alone, which is valid for BPRS as well. Even though an efficiency comparison shows 78.39 and 83.54 percent for BPR and BPRS respectively, from this study it can be seen that there is around 22.01 percent of BPRs capable of being very efficient in carrying out the intermediation process. Whereas for BPRS, around 25.49 percent are able to achieve similar results.

**Table 4:** The classification of BPR and BPRS according to the technical efficiency results.

Institution	Classification	Period									
		2013		2014		2015		2016		2017	
		Unit	Percent	Unit	Percent	Unit	Percent	Unit	Percent	Unit	Percent
**BPRS**											
Intermediation Approach	Very Efficient	13	11.50	8	7.08	7	6.19	9	7.96	9	7.96
	Efficient	19	16.81	19	16.81	19	16.81	20	17.70	21	18.58
	Quite Efficient	18	15.93	20	17.70	22	19.47	19	16.81	23	20.35
	Inefficient	22	19.47	23	20.35	23	20.35	22	19.47	18	15.93
	Very Inefficient	41	36.28	43	38.05	42	37.17	43	38.05	42	37.17
Production Approach	Very Efficient	41	36.28	40	35.40	41	36.28	41	36.28	43	38.05
	Efficient	29	25.66	46	40.71	57	50.44	64	56.64	70	61.95
	Quite Efficient	23	20.35	22	19.47	15	13.27	8	7.08	0	0.00
	Inefficient	14	12.39	5	4.42	0	0.00	0	0.00	0	0.00
	Very Inefficient	6	5.31	0	0.00	0	0.00	0	0.00	0	0.00
Total Samples		113									

Comparison of the two institutions using DEA has been conducted in several studies, but the variables and amounts used are not as complex as this research. Interestingly, the efficiency of BPRS is higher than BPR (Muhari and Hosen, 2014; Putri, 2016; Zuhroh et al., 2015). According to Hartono et al. (2008) BPRs have been inefficient since 2005 (Hartono et al., 2008). In some regions, the same results are also shown, specifically, efficient BPRs are less than those that are inefficient, such as in Semarang (Septianto and Widiharih, 2010) and Jakarta-Bogor-Depok-Tangerang-Bekasi (JABODETABEK) (Hartono et al., 2008). The average technical efficiency of BPR that is lower than BPRS may be due to the number of cases of fraud in BPR that are very serious (Rustiarini et al., 2016). As a result of this fraud, the community is increasingly distrustful of BPRs, which has resulted in a decline in their performance.

There are conventional and Islamic banks in other countries that actually show different results from Indonesia, for instance, in Malaysia, each bank has a different type of efficiency. Islamic banks are considered more capable of allocating and utilizing their resources, while conventional banks are more efficient because they utilize information and electronic technology (Ismail et al., 2013). In the Middle East, Islamic banks are less efficient than conventional (Rosman et al., 2014).

After calculating the efficiency level of each BPR and BPRS, it is important to determine the factors that influence their intermediation and production efficiencies. Generally, Table 5 shows the condition of the variables used in the Tobit model. From 2013 to 2017, the average CAR, business scale and volume of BPR and BPRS were not significantly different. However, a striking difference exists in the condition of non-performing loans where the level of BPRS NPL is much higher than BPR. Although not significantly different, the average BPRS ownership by local governments tends to be higher. This indicates their tendency to switch to the Islamic financial system in managing their finances. In addition, the average BPRS located in cities is also higher.

**Table 5:** Descriptive statistics of variables used in the Tobit model.

Variables	Mean	Std. Dev	Min	Max
**BPR**				
TE-Intermediation	0.51	0.29	0.00	1.00
TE-Production	0.86	0.15	0.20	1.00
CAR	0.20	0.34	0.01	8.06
NPL	0.08	0.38	0.01	18.00
Owner	0.07	0.26	0.00	1.00
Loc	0.25	0.43	0.00	1.00
Size	17.01	1.23	12.55	22.21
Turnover	15.44	1.15	9.71	20.67
**BPRS**				
TE-Intermediation	0.53	0.31	0.01	1.00
TE-Production	0.89	0.15	0.20	1.00
CAR	0.21	0.29	0.01	4.35
NPL	10.81	11.62	0.01	85.07
Owner	0.19	0.39	0.00	1.00
Loc	0.33	0.47	0.00	1.00
Size	17.05	1.17	13.31	20.78
Turnover	15.32	1.11	11.70	19.06

The data summarized in Table 5 are then processed using the Tobit model to determine which factors affects the level of technical efficiency using both intermediation and production approaches. Overall, the results of the Tobit estimation in this study can be shown in Table 6.

**Table 6:** Tobit estimation results.

Variables	Intermediation		Production	
	BPR	BPRS	BPR	BPRS
car	0.015663	0.043206	0.008595	0.0672581
	0.0016808∗∗∗	0.0170889∗∗	0.0054259	0.0251003∗∗∗
NPL/NPF	0.0002775	-0.0006286	0.0018252	0.0010656
	0.0012219	0.000372∗	0.0048	0.0005653∗
ldr	2.80e-06	-3.71e-06	-0.0002546	0.000046
	0.0000201	0.0001278	0.0000831∗∗∗	0.0001813
owner	0.0078902	-0.0844014	0.0057572	-0.0269201
	0.019017	0.0712616	0.0070569	0.0191252
loc	0.4299951	0.1047641	0.1237972	0.0349064
	0.0146986∗∗∗	0.0473664∗∗	0.0042312∗∗∗	0.0153156∗∗
size	0.0008238	-0.0077431	0.0010532	0.0294696
	0.0005161	0.0089912	0.001502	0.0071274∗∗∗
constant	0.3831199	0.6449112	0.824713	0.351693
	0.0117142∗∗∗	0.1590406∗∗∗	0.0264482∗∗∗	0.1240744∗∗∗
Wald chi2	949.79∗∗∗	20.80∗∗∗	892.75∗∗∗	27.87∗∗∗

Note: ∗∗∗ significant at 1%, ∗∗ significant at 5%, ∗ significant at 10%.

From Table 6, it can be seen that the capital adequacy ratio (CAR) consistently has a significant effect on the intermediation efficiency of both BPR and BPRS, and the production efficiency of BPRS. It cannot be denied that the capital adequacy of the two institutions greatly determines their financial performance. The greater the capital owned, the higher the level of production and intermediation efficiency. CAR shows the ability of BPR and BPRS in providing funds to anticipate the possibility of default. When CAR increases mainly due to the higher capital and or low-risk assets, the potential to achieve efficiency in both production and intermediation will also be higher. High capital is basically a source of cheap funds for banks, therefore, the selling price of their credit will be more competitive. Of course, this makes bank intermediation capabilities higher because these funds are deposited funds that have a small possibility of being withdrawn by investors except in a state of dispute or bankruptcy. This finding is consistent with the research conducted by Anwar (2016) which states that CAR is one of the important components in improving banking efficiency (Anwar, 2016).

Non-performing loans only affect the intermediation and production efficiencies of BPRS, despite a 10 percent significance level. Similar to Devi and Firmansyah (2018), we also found on the high NPF has a negative impact on the efficiency of the collection and distribution of funds (Devi and Firmansyah, 2018). BPRS seems to be increasingly careful in channeling funds because it is feared that this will lead to higher risks in the future. However, NPLs actually have a positive effect on the institution's production efficiency. The average NPL of BPRS continues to increase gradually from 9.06 percent in 2013 to 12.01 percent in 2017, while the level of production efficiency also rises from 0.80 to 0.95. Increasing the prudence of BPRS in the distribution of finance may improve its quality. Thus, making it possible to obtain relatively high income from good quality customers.

The loan to deposit ratio has a significant negative effect on the level of efficiency of BPR production. The higher the LDR level, the lower the score. This is likely to happen when the credit characteristics of the BPR are quite risky. Thus, to maintain production performance, it should regulate its LDR in a safe position.

The locations in which BPRs and BPRS operate have a significant role in influencing the level of production efficiency and intermediation. In this study, the aspect of location was attempted to be included as a determinant of efficiency scores. This variable was chosen to determine whether the location of the BPR or BPRS in the Municipality and District was different considering that the Municipality was identical to the area that had more complete facilities and infrastructure as well as a higher level of community income. Although on average, there are many in the Regency region, it follows that BPRs and BPRS located in the Municipality have a higher chance of being more efficient. Location factors turned out to be more positively influential on intermediation efficiency. This means that there are more opportunities for BPR and BPRS to collect and channel funds to the city community than other regions. However, although significant, the effect of location on production efficiency is not as large as intermediation. Thus, obtaining profits in the Regency region also has equally small opportunities compared to BPRs and BPRS operating in the Municipality area.

The business scale has a significant positive effect on the production efficiency score of BPRS. As a relatively new player in the Indonesian banking industry, high assets greatly contribute to increasing production efficiency. In this case, it is possible that the operational costs incurred are also relatively low. Supposing this study also shows that CAR and assets have a significant positive effect on BPRS production efficiency scores, then it should increase asset components other than capital, for example, third-party funds or those from Sharia commercial banks.

Previous research stated that there are several causes of efficient and inefficient BPR and BPRS. For a number of cases, BPRs that carry out mergers are fully efficient (Hartono et al., 2008). The causes of inefficiencies are capital, third-party funds, and excessive interest expenses when lending and bank interest income are less than optimal (Putri, 2016). In BPRS, the causes of inefficiency are the absence of financing that contains profit sharing and business competition between Islamic microfinance institutions (Fauzi, 2014), and low ROA, ROE, and liquidity (Hamidi, 2017). In order to avoid these, some efforts are needed such as controlling other income variables, current assets, total fixed assets, third-party funds, and workforce expenses (Muhari and Hosen, 2014; Sembiring, 2019). BPRS that operate with sharia principles can optimize transactions or contracts that use profit-sharing contracts and control their assets and liquidity, reduce production costs (Miah and Uddin, 2017), and increase bank size (S. Ahmad et al., 2012).

## 5 Conclusion

From the technical efficacy calculation using the DEA method, this study concludes that BPRs and BPRS are still inefficient in carrying out their intermediary roles. However, both institutions have been proven efficient in the production aspect. To improve the efficiency of intermediation and production, both institutions should increase their capital. This is because, from the results of Tobit's estimation, the capital adequacy ratio has a significant positive effect on technical efficiency in both approaches. Additionally, the location factor also has an influence as it can be seen that the more there is in the city, the greater the potential for efficiency. Evidently, the city has a more complete infrastructure that allows for quicker business development.

The overall efficiency associated with the production and intermediation of BPRS is relatively slightly higher compared to BPR (Anwar, 2016), which shows that its financial performance is better. This is certainly in line with the number of BPRs liquidated by LPSwhich does not make it feel safe due to the 1.2 liquidated of the younger age differences leaving4 units or 2.2 percent currently in the liquidation process.

Although Tobit regression estimation has been conducted to look for factors capable of affecting the efficiency of BPR and BPRS, The model in this study does not cover several other important causes such as the existence of banking crimes which turned out to be the main cause of the default of many BPRs in Indonesia (Rustiarini et al., 2016). The various forms of competition between banks, product innovations and other financial institutions which also targets microfinance, and innovation technology in financial worlds such as fintech. This became a limitation of this study which is important to highlight due to the efficiency of BPR and BPRS which is not solely determined by the variables analyzed in the Tobit model.

Furthermore, from this study, it appears that there was a trade-off between the functions of production and intermediation in both BPRs and BPRS as microfinance institutions. The inefficiency of their intermediation aspects turned out to be accompanied by the success of production. This certainly raises a new question; Does this really have to be the case in microfinance institutions in Indonesia? To maintain financial sustainability, the companies limited the distribution of funds and chose healthy partners, therefore, they succeeded in making efficient profits.

Then, the next questions arise; What is the profile of recipients of BPR and BPRS funds? Are microfinance institutions intended to develop micro, small and medium enterprises? If not, which institutions will serve the businesses not covered by BPRs or BPRS? This is certainly a recommendation for further research to answer these questions. The Financial Services Authority or Otoritas Jasa Keuangan (OJK) should provide more supervision of inefficient BPRs and BPRS in order not to disrupt the stability of the banking industry in Indonesia.

## Declarations

### Author contribution statement

Wasiaturrahma and S.R. Ajija: Conceived and designed the experiments; Performed the experiments; Analyzed and interpreted the data; Contributed reagents, materials, analysis tools or data; Wrote the paper.

R. Sukmana: Performed the experiments; Contributed reagents, materials, analysis tools or data; Wrote the paper.

S.C.U. Salama: Performed the experiments; Wrote the paper.

A. Hudaifah: Contributed reagents, materials, analysis tools or data; Wrote the paper.

### Funding statement

This research is funded by the Indonesia Ministry of Research, Technology, & Higher Education.

### Competing interest statement

The authors declare no conflict of interest.

### Additional information

No additional information is available for this paper.

## Appendix A Supplementary data

Calculating Outreach Index Calculating Outreach Index
