# Supplementary material for: Financial performance of rural banks in Indonesia: A two-stage DEA approach
Source: Heliyon. 2020 Jul 17;6(7):e04390. doi: 10.1016/j.heliyon.2020.e04390 (PMC7371765; doi:10.1016/j.heliyon.2020.e04390)
Supplement: Calculating Outreach Index [file mmc1.docx]

**Appendix**

| **KSPPS MUI** | |  |  |  |  |  |  |  |  |  |  |  |
| --- | --- | --- | --- | --- | --- | --- | --- | --- | --- | --- | --- | --- |
| Outreach | Indicator | Scale | 2014 | | 2015 | | 2016 | | 2017 | | 2018 | |
|  |  |  | Data | Score | Data | Score | Data | Score | Data | Score | Data | Score |
| Breadth | The Number of Financing Receiver | 0 🡪 < 20.000 | 45 | 0 | 52 | 0 | 70 | 0 | 103 | 0 | 170 | 0 |
|  |  | 1 🡪 20.000 – 50.000 |  |  |  |  |  |  |  |  |  |  |
|  |  | 2 🡪 > 50.000 |  |  |  |  |  |  |  |  |  |  |
|  | The percentage of financial aid recipients used for non-productive activities | 0 🡪 < 10% | 45 | 2 | 43 | 2 | 35 | 2 | 25 | 1 | 20 | 1 |
|  |  | 1 🡪 10% - 30% |  |  |  |  |  |  |  |  |  |  |
|  |  | 2 🡪 > 30% |  |  |  |  |  |  |  |  |  |  |
|  | Percentage of voluntary savers compared to total recipients of the fund | 0 🡪 < 50% | 207 | 2 | 183 | 2 | 179 | 2 | 164 | 2 | 131 | 2 |
|  |  | 1 🡪 50% - 75% |  |  |  |  |  |  |  |  |  |  |
|  |  | 2 🡪 > 75% |  |  |  |  |  |  |  |  |  |  |
|  | Percentage of members who that use MFI services such as transfers or insurance to the total recipient of the fund | 0 🡪 < 10% | 0 | 0 | 0 | 0 | 0 | 0 | 0 | 0 | 0 | 0 |
|  |  | 1 🡪 10% - 30% |  |  |  |  |  |  |  |  |  |  |
|  |  | 2 🡪 > 30% |  |  |  |  |  |  |  |  |  |  |
|  | Percentage of members that received non-financial facilities such as training or other empowerment programs to the total recipient of the fund | 0 🡪 < 10% | 0 | 0 | 0 | 0 | 0 | 0 | 0 | 0 | 0 | 0 |
|  |  | 1 🡪 10% - 30% |  |  |  |  |  |  |  |  |  |  |
|  |  | 2 🡪 > 30% |  |  |  |  |  |  |  |  |  |  |
| Total | | |  | 4 |  | 4 |  | 4 |  | 3 |  | 3 |
| Breadth Outreach Score (Total Score : 10) | | |  | 0.4 |  | 0.4 |  | 0.4 |  | 0.3 |  | 0.3 |
| Depth | The average percentage of funding provided to members per Gross Domestic Regional Product per capita in East Java | 0 🡪 > 100% | 132 | 0 | 177 | 0 | 191 | 0 | 207 | 0 | 187 | 0 |
|  |  | 1 🡪 60% - 100% |  |  |  |  |  |  |  |  |  |  |
|  |  | 2 🡪 < 60% |  |  |  |  |  |  |  |  |  |  |
|  | Percentage of financing under $ 300 (equivalent to Rp.4,027,500) | 0 🡪 < 20% | 55 | 2 | 50 | 1 | 45 | 1 | 40 | 1 | 40 | 1 |
|  |  | 1 🡪 20% - 50% |  |  |  |  |  |  |  |  |  |  |
|  |  | 2 🡪 > 50% |  |  |  |  |  |  |  |  |  |  |
|  | Percentage of number of female financing recipients | 0 🡪 < 20% | 35 | 1 | 37 | 1 | 40 | 1 | 36 | 1 | 30 | 1 |
|  |  | 1 🡪 20% - 50% |  |  |  |  |  |  |  |  |  |  |
|  |  | 2 🡪 > 50% |  |  |  |  |  |  |  |  |  |  |
|  | Percentage of recipients of funding from villages | 0 🡪 < 15% | 100 | 2 | 100 | 2 | 100 | 2 | 90 | 2 | 85 | 2 |
|  |  | 1 🡪 15% - 30% |  |  |  |  |  |  |  |  |  |  |
|  |  | 2 🡪 > 30% |  |  |  |  |  |  |  |  |  |  |
|  | Percentage of financing to entrepreneurs originating from poor customers targeted by the poverty alleviation programs | 0 🡪 < 20% | 15 | 0 | 15 | 0 | 10 | 0 | 10 | 0 | 5 | 0 |
|  |  | 1 🡪 20% - 50% |  |  |  |  |  |  |  |  |  |  |
|  |  | 2 🡪 > 50% |  |  |  |  |  |  |  |  |  |  |
| Total | | |  | 5 |  | 4 |  | 4 |  | 4 |  | 4 |
| Depth Outreach Score (Total Score : 10) | | |  | 0.5 |  | 0.4 |  | 0.4 |  | 0.4 |  | 0.4 |
| Total of Outreach Score (Total of Breadth and Depth Score) | | |  | 9 |  | 8 |  | 8 |  | 7 |  | 7 |
| Overall Outreach Score (Total of Outreach Score : 20) | | |  | 0.45 |  | 0.4 |  | 0.4 |  | 0.35 |  | 0.35 |

| **BMT Permata** | |  |  |  |  |  |  |  |  |  |  |  |
| --- | --- | --- | --- | --- | --- | --- | --- | --- | --- | --- | --- | --- |
| Outreach | Indicator | Scale | 2014 | | 2015 | | 2016 | | 2017 | | 2018 | |
|  |  |  | Data | Score | Data | Score | Data | Score | Data | Score | Data | Score |
| Breadth | The Number of Financing Receiver | 0 🡪 < 20.000 | 64 | 0 | 81 | 0 | 78 | 0 | 126 | 0 | 198 | 0 |
|  |  | 1 🡪 20.000 – 50.000 |  |  |  |  |  |  |  |  |  |  |
|  |  | 2 🡪 > 50.000 |  |  |  |  |  |  |  |  |  |  |
|  | The percentage of financial aid recipients used for non-productive activities | 0 🡪 < 10% | 28 | 1 | 26 | 1 | 32 | 2 | 34 | 2 | 41 | 2 |
|  |  | 1 🡪 10% - 30% |  |  |  |  |  |  |  |  |  |  |
|  |  | 2 🡪 > 30% |  |  |  |  |  |  |  |  |  |  |
|  | Percentage of voluntary savers compared to total recipients of the fund | 0 🡪 < 50% | 136 | 2 | 170 | 2 | 171 | 2 | 133 | 2 | 132 | 2 |
|  |  | 1 🡪 50% - 75% |  |  |  |  |  |  |  |  |  |  |
|  |  | 2 🡪 > 75% |  |  |  |  |  |  |  |  |  |  |
|  | Percentage of members who that use MFI services such as transfers or insurance to the total recipient of the fund | 0 🡪 < 10% | 75 | 2 | 82 | 2 | 78 | 2 | 86 | 2 | 87 | 2 |
|  |  | 1 🡪 10% - 30% |  |  |  |  |  |  |  |  |  |  |
|  |  | 2 🡪 > 30% |  |  |  |  |  |  |  |  |  |  |
|  | Percentage of members that received non-financial facilities such as training or other empowerment programs to the total recipient of the fund | 0 🡪 < 10% | 22 | 1 | 28 | 1 | 33 | 2 | 30 | 1 | 34 | 2 |
|  |  | 1 🡪 10% - 30% |  |  |  |  |  |  |  |  |  |  |
|  |  | 2 🡪 > 30% |  |  |  |  |  |  |  |  |  |  |
| Total | | |  | 6 |  | 6 |  | 8 |  | 7 |  | 8 |
| Breadth Outreach Score (Total Score : 10) | | |  | 0.6 |  | 0.6 |  | 0.8 |  | 0.7 |  | 0.8 |
| Depth | The average percentage of funding provided to members per Gross Domestic Regional Product per capita in East Java | 0 🡪 > 100% | 89 | 1 | 98 | 1 | 98 | 1 | 78 | 1 | 51 | 2 |
|  |  | 1 🡪 60% - 100% |  |  |  |  |  |  |  |  |  |  |
|  |  | 2 🡪 < 60% |  |  |  |  |  |  |  |  |  |  |
|  | Percentage of financing under $ 300 (equivalent to Rp.4,027,500) | 0 🡪 < 20% | 35 | 1 | 27 | 1 | 17 | 0 | 8 | 0 | 8 | 0 |
|  |  | 1 🡪 20% - 50% |  |  |  |  |  |  |  |  |  |  |
|  |  | 2 🡪 > 50% |  |  |  |  |  |  |  |  |  |  |
|  | Percentage of number of female financing recipients | 0 🡪 < 20% | 47 | 1 | 50 | 1 | 43 | 1 | 40 | 1 | 42 | 1 |
|  |  | 1 🡪 20% - 50% |  |  |  |  |  |  |  |  |  |  |
|  |  | 2 🡪 > 50% |  |  |  |  |  |  |  |  |  |  |
|  | Percentage of recipients of funding from villages | 0 🡪 < 15% | 46 | 2 | 43 | 2 | 48 | 2 | 51 | 2 | 49 | 2 |
|  |  | 1 🡪 15% - 30% |  |  |  |  |  |  |  |  |  |  |
|  |  | 2 🡪 > 30% |  |  |  |  |  |  |  |  |  |  |
|  | Percentage of financing to entrepreneurs originating from poor customers targeted by the poverty alleviation programs | 0 🡪 < 20% | 12 | 0 | 15 | 0 | 23 | 1 | 32 | 1 | 28 | 1 |
|  |  | 1 🡪 20% - 50% |  |  |  |  |  |  |  |  |  |  |
|  |  | 2 🡪 > 50% |  |  |  |  |  |  |  |  |  |  |
| Total | | |  | 5 |  | 5 |  | 5 |  | 5 |  | 6 |
| Depth Outreach Score (Total Score : 10) | | |  | 0.5 |  | 0.5 |  | 0.5 |  | 0.5 |  | 0.6 |
| Total of Outreach Score (Total of Breadth and Depth Score) | | |  | 11 |  | 11 |  | 13 |  | 12 |  | 14 |
| Overall Outreach Score (Total of Outreach Score : 20) | | |  | 0.55 |  | 0.55 |  | 0.65 |  | 0.6 |  | 0.7 |

| **BMT Muda** | |  |  |  |  |  |  |  |  |  |  |  |
| --- | --- | --- | --- | --- | --- | --- | --- | --- | --- | --- | --- | --- |
| Outreach | Indicator | Scale | 2014 | | 2015 | | 2016 | | 2017 | | 2018 | |
|  |  |  | Data | Score | Data | Score | Data | Score | Data | Score | Data | Score |
| Breadth | The Number of Financing Receiver | 0 🡪 < 20.000 | 132 | 0 | 156 | 0 | 235 | 0 | 250 | 0 | 259 | 0 |
|  |  | 1 🡪 20.000 – 50.000 |  |  |  |  |  |  |  |  |  |  |
|  |  | 2 🡪 > 50.000 |  |  |  |  |  |  |  |  |  |  |
|  | The percentage of financial aid recipients used for non-productive activities | 0 🡪 < 10% | 20 | 1 | 17 | 1 | 15 | 1 | 13 | 1 | 13 | 1 |
|  |  | 1 🡪 10% - 30% |  |  |  |  |  |  |  |  |  |  |
|  |  | 2 🡪 > 30% |  |  |  |  |  |  |  |  |  |  |
|  | Percentage of voluntary savers compared to total recipients of the fund | 0 🡪 < 50% | 189 | 2 | 169 | 2 | 183 | 2 | 222 | 2 | 225 | 2 |
|  |  | 1 🡪 50% - 75% |  |  |  |  |  |  |  |  |  |  |
|  |  | 2 🡪 > 75% |  |  |  |  |  |  |  |  |  |  |
|  | Percentage of members who that use MFI services such as transfers or insurance to the total recipient of the fund | 0 🡪 < 10% | 52 | 2 | 58 | 2 | 62 | 2 | 63 | 2 | 65 | 2 |
|  |  | 1 🡪 10% - 30% |  |  |  |  |  |  |  |  |  |  |
|  |  | 2 🡪 > 30% |  |  |  |  |  |  |  |  |  |  |
|  | Percentage of members that received non-financial facilities such as training or other empowerment programs to the total recipient of the fund | 0 🡪 < 10% | 15 | 1 | 16 | 1 | 20 | 1 | 26 | 1 | 30 | 2 |
|  |  | 1 🡪 10% - 30% |  |  |  |  |  |  |  |  |  |  |
|  |  | 2 🡪 > 30% |  |  |  |  |  |  |  |  |  |  |
| Total | | |  | 6 |  | 6 |  | 6 |  | 6 |  | 7 |
| Breadth Outreach Score (Total Score : 10) | | |  | 0.6 |  | 0.6 |  | 0.6 |  | 0.6 |  | 0.7 |
| Depth | The average percentage of funding provided to members per Gross Domestic Regional Product per capita in East Java | 0 🡪 > 100% | 25 | 2 | 33 | 2 | 23 | 2 | 17 | 2 | 16 | 2 |
|  |  | 1 🡪 60% - 100% |  |  |  |  |  |  |  |  |  |  |
|  |  | 2 🡪 < 60% |  |  |  |  |  |  |  |  |  |  |
|  | Percentage of financing under $ 300 (equivalent to Rp.4,027,500) | 0 🡪 < 20% | 25 | 1 | 20 | 1 | 18 | 0 | 15 | 0 | 12 | 0 |
|  |  | 1 🡪 20% - 50% |  |  |  |  |  |  |  |  |  |  |
|  |  | 2 🡪 > 50% |  |  |  |  |  |  |  |  |  |  |
|  | Percentage of number of female financing recipients | 0 🡪 < 20% | 87 | 2 | 85 | 2 | 83 | 2 | 80 | 2 | 75 | 2 |
|  |  | 1 🡪 20% - 50% |  |  |  |  |  |  |  |  |  |  |
|  |  | 2 🡪 > 50% |  |  |  |  |  |  |  |  |  |  |
|  | Percentage of recipients of funding from villages | 0 🡪 < 15% | 60 | 2 | 63 | 2 | 64 | 2 | 68 | 2 | 70 | 2 |
|  |  | 1 🡪 15% - 30% |  |  |  |  |  |  |  |  |  |  |
|  |  | 2 🡪 > 30% |  |  |  |  |  |  |  |  |  |  |
|  | Percentage of financing to entrepreneurs originating from poor customers targeted by the poverty alleviation programs | 0 🡪 < 20% | 6.6 | 0 | 6.4 | 0 | 6.3 | 0 | 6.7 | 0 | 6.5 | 1 |
|  |  | 1 🡪 20% - 50% |  |  |  |  |  |  |  |  |  |  |
|  |  | 2 🡪 > 50% |  |  |  |  |  |  |  |  |  |  |
| Total | | |  | 7 |  | 7 |  | 6 |  | 6 |  | 6 |
| Depth Outreach Score (Total Score : 10) | | |  | 0.7 |  | 0.7 |  | 0.6 |  | 0.6 |  | 0.6 |
| Total of Outreach Score (Total of Breadth and Depth Score) | | |  | 13 |  | 13 |  | 12 |  | 12 |  | 13 |
| Overall Outreach Score (Total of Outreach Score : 20) | | |  | 0.65 |  | 0.65 |  | 0.6 |  | 0.6 |  | 0.65 |

| **BMT Al-Izzah** | |  |  |  |  |  |  |  |  |  |  |  |
| --- | --- | --- | --- | --- | --- | --- | --- | --- | --- | --- | --- | --- |
| Outreach | Indicator | Scale | 2014 | | 2015 | | 2016 | | 2017 | | 2018 | |
|  |  |  | Data | Score | Data | Score | Data | Score | Data | Score | Data | Score |
| Breadth | The Number of Financing Receiver | 0 🡪 < 20.000 | 465 | 0 | 343 | 0 | 466 | 0 | 530 | 0 | 510 | 0 |
|  |  | 1 🡪 20.000 – 50.000 |  |  |  |  |  |  |  |  |  |  |
|  |  | 2 🡪 > 50.000 |  |  |  |  |  |  |  |  |  |  |
|  | The percentage of financial aid recipients used for non-productive activities | 0 🡪 < 10% | 29.3 | 1 | 79.6 | 2 | 96.7 | 2 | 93.1 | 2 | 92.5 | 2 |
|  |  | 1 🡪 10% - 30% |  |  |  |  |  |  |  |  |  |  |
|  |  | 2 🡪 > 30% |  |  |  |  |  |  |  |  |  |  |
|  | Percentage of voluntary savers compared to total recipients of the fund | 0 🡪 < 50% | 109 | 2 | 120 | 2 | 101 | 2 | 108 | 2 | 116 | 2 |
|  |  | 1 🡪 50% - 75% |  |  |  |  |  |  |  |  |  |  |
|  |  | 2 🡪 > 75% |  |  |  |  |  |  |  |  |  |  |
|  | Percentage of members who that use MFI services such as transfers or insurance to the total recipient of the fund | 0 🡪 < 10% | 80 | 2 | 90 | 2 | 90 | 2 | 90 | 2 | 90 | 2 |
|  |  | 1 🡪 10% - 30% |  |  |  |  |  |  |  |  |  |  |
|  |  | 2 🡪 > 30% |  |  |  |  |  |  |  |  |  |  |
|  | Percentage of members that received non-financial facilities such as training or other empowerment programs to the total recipient of the fund | 0 🡪 < 10% | 30 | 1 | 30 | 1 | 30 | 1 | 30 | 1 | 30 | 1 |
|  |  | 1 🡪 10% - 30% |  |  |  |  |  |  |  |  |  |  |
|  |  | 2 🡪 > 30% |  |  |  |  |  |  |  |  |  |  |
| Total | | |  | 6 |  | 7 |  | 7 |  | 7 |  | 7 |
| Breadth Outreach Score (Total Score : 10) | | |  | 0.6 |  | 0.7 |  | 0.7 |  | 0.7 |  | 0.7 |
| Depth | The average percentage of funding provided to members per Gross Domestic Regional Product per capita in East Java | 0 🡪 > 100% | 8 | 2 | 10 | 2 | 9 | 2 | 7 | 2 | 8 | 2 |
|  |  | 1 🡪 60% - 100% |  |  |  |  |  |  |  |  |  |  |
|  |  | 2 🡪 < 60% |  |  |  |  |  |  |  |  |  |  |
|  | Percentage of financing under $ 300 (equivalent to Rp.4,027,500) | 0 🡪 < 20% | 85 | 2 | 85 | 2 | 85 | 2 | 85 | 2 | 90 | 2 |
|  |  | 1 🡪 20% - 50% |  |  |  |  |  |  |  |  |  |  |
|  |  | 2 🡪 > 50% |  |  |  |  |  |  |  |  |  |  |
|  | Percentage of number of female financing recipients | 0 🡪 < 20% | 55 | 2 | 50 | 1 | 45 | 1 | 60 | 2 | 45 | 1 |
|  |  | 1 🡪 20% - 50% |  |  |  |  |  |  |  |  |  |  |
|  |  | 2 🡪 > 50% |  |  |  |  |  |  |  |  |  |  |
|  | Percentage of recipients of funding from villages | 0 🡪 < 15% | 55 | 2 | 55 | 2 | 55 | 2 | 55 | 2 | 55 | 2 |
|  |  | 1 🡪 15% - 30% |  |  |  |  |  |  |  |  |  |  |
|  |  | 2 🡪 > 30% |  |  |  |  |  |  |  |  |  |  |
|  | Percentage of financing to entrepreneurs originating from poor customers targeted by the poverty alleviation programs | 0 🡪 < 20% | 84.08 | 2 | 63.26 | 2 | 83.5 | 2 | 75.48 | 2 | 78.44 | 2 |
|  |  | 1 🡪 20% - 50% |  |  |  |  |  |  |  |  |  |  |
|  |  | 2 🡪 > 50% |  |  |  |  |  |  |  |  |  |  |
| Total | | |  | 10 |  | 9 |  | 9 |  | 10 |  | 9 |
| Depth Outreach Score (Total Score : 10) | | |  | 1 |  | 0.9 |  | 0.9 |  | 1 |  | 0.9 |
| Total of Outreach Score (Total of Breadth and Depth Score) | | |  | 16 |  | 16 |  | 16 |  | 17 |  | 16 |
| Overall Outreach Score (Total of Outreach Score : 20) | | |  | 0.8 |  | 0.8 |  | 0.8 |  | 0.85 |  | 0.85 |

| **Kanindo Syariah** | |  |  |  |  |  |  |  |  |  |  |  |
| --- | --- | --- | --- | --- | --- | --- | --- | --- | --- | --- | --- | --- |
| Outreach | Indicator | Scale | 2014 | | 2015 | | 2016 | | 2017 | | 2018 | |
|  |  |  | Data | Score | Data | Score | Data | Score | Data | Score | Data | Score |
| Breadth | The Number of Financing Receiver | 0 🡪 < 20.000 | 15406 | 0 | 15779 | 0 | 20112 | 1 | 25486 | 1 | 33504 | 1 |
|  |  | 1 🡪 20.000 – 50.000 |  |  |  |  |  |  |  |  |  |  |
|  |  | 2 🡪 > 50.000 |  |  |  |  |  |  |  |  |  |  |
|  | The percentage of financial aid recipients used for non-productive activities | 0 🡪 < 10% | 0.1 | 0 | 0.2 | 0 | 0.3 | 0 | 0.5 | 0 | 0.5 | 0 |
|  |  | 1 🡪 10% - 30% |  |  |  |  |  |  |  |  |  |  |
|  |  | 2 🡪 > 30% |  |  |  |  |  |  |  |  |  |  |
|  | Percentage of voluntary savers compared to total recipients of the fund | 0 🡪 < 50% | 136 | 2 | 132 | 2 | 128 | 2 | 122 | 2 | 117 | 2 |
|  |  | 1 🡪 50% - 75% |  |  |  |  |  |  |  |  |  |  |
|  |  | 2 🡪 > 75% |  |  |  |  |  |  |  |  |  |  |
|  | Percentage of members who that use MFI services such as transfers or insurance to the total recipient of the fund | 0 🡪 < 10% | 0.3 | 0 | 0.3 | 0 | 0.5 | 0 | 0.1 | 0 | 0.1 | 0 |
|  |  | 1 🡪 10% - 30% |  |  |  |  |  |  |  |  |  |  |
|  |  | 2 🡪 > 30% |  |  |  |  |  |  |  |  |  |  |
|  | Percentage of members that received non-financial facilities such as training or other empowerment programs to the total recipient of the fund | 0 🡪 < 10% | 0.1 | 0 | 0.1 | 0 | 0.1 | 0 | 0.2 | 0 | 0.2 | 0 |
|  |  | 1 🡪 10% - 30% |  |  |  |  |  |  |  |  |  |  |
|  |  | 2 🡪 > 30% |  |  |  |  |  |  |  |  |  |  |
| Total | | |  | 2 |  | 2 |  | 3 |  | 3 |  | 3 |
| Breadth Outreach Score (Total Score : 10) | | |  | 0.2 |  | 0.2 |  | 0.3 |  | 0.3 |  | 0.3 |
| Depth | The average percentage of funding provided to members per Gross Domestic Regional Product per capita in East Java | 0 🡪 > 100% | 5 | 2 | 4 | 2 | 3 | 2 | 2 | 2 | 2 | 2 |
|  |  | 1 🡪 60% - 100% |  |  |  |  |  |  |  |  |  |  |
|  |  | 2 🡪 < 60% |  |  |  |  |  |  |  |  |  |  |
|  | Percentage of financing under $ 300 (equivalent to Rp.4,027,500) | 0 🡪 < 20% | 50 | 1 | 50 | 1 | 45 | 1 | 45 | 1 | 50 | 1 |
|  |  | 1 🡪 20% - 50% |  |  |  |  |  |  |  |  |  |  |
|  |  | 2 🡪 > 50% |  |  |  |  |  |  |  |  |  |  |
|  | Percentage of number of female financing recipients | 0 🡪 < 20% | 15 | 0 | 18 | 0 | 20 | 1 | 20 | 1 | 25 | 1 |
|  |  | 1 🡪 20% - 50% |  |  |  |  |  |  |  |  |  |  |
|  |  | 2 🡪 > 50% |  |  |  |  |  |  |  |  |  |  |
|  | Percentage of recipients of funding from villages | 0 🡪 < 15% | 80 | 2 | 80 | 2 | 80 | 2 | 80 | 2 | 80 | 2 |
|  |  | 1 🡪 15% - 30% |  |  |  |  |  |  |  |  |  |  |
|  |  | 2 🡪 > 30% |  |  |  |  |  |  |  |  |  |  |
|  | Percentage of financing to entrepreneurs originating from poor customers targeted by the poverty alleviation programs | 0 🡪 < 20% | 0 | 0 | 0 | 0 | 0 | 0 | 0 | 0 | 0 | 0 |
|  |  | 1 🡪 20% - 50% |  |  |  |  |  |  |  |  |  |  |
|  |  | 2 🡪 > 50% |  |  |  |  |  |  |  |  |  |  |
| Total | | |  | 5 |  | 5 |  | 6 |  | 6 |  | 6 |
| Depth Outreach Score (Total Score : 10) | | |  | 0.5 |  | 0.5 |  | 0.6 |  | 0.6 |  | 0.6 |
| Total of Outreach Score (Total of Breadth and Depth Score) | | |  | 7 |  | 7 |  | 9 |  | 9 |  | 9 |
| Overall Outreach Score (Total of Outreach Score : 20) | | |  | 0.35 |  | 0.35 |  | 0.45 |  | 0.45 |  | 0.45 |

| **KSPPS DMU Jatim** | |  |  |  |  |  |  |  |  |  |  |  |
| --- | --- | --- | --- | --- | --- | --- | --- | --- | --- | --- | --- | --- |
| Outreach | Indicator | Scale | 2014 | | 2015 | | 2016 | | 2017 | | 2018 | |
|  |  |  | Data | Score | Data | Score | Data | Score | Data | Score | Data | Score |
| Breadth | The Number of Financing Receiver | 0 🡪 < 20.000 | 200 | 0 | 377 | 0 | 522 | 0 | 632 | 0 | 872 | 0 |
|  |  | 1 🡪 20.000 – 50.000 |  |  |  |  |  |  |  |  |  |  |
|  |  | 2 🡪 > 50.000 |  |  |  |  |  |  |  |  |  |  |
|  | The percentage of financial aid recipients used for non-productive activities | 0 🡪 < 10% | 5 | 0 | 7 | 0 | 16 | 1 | 19 | 1 | 29 | 1 |
|  |  | 1 🡪 10% - 30% |  |  |  |  |  |  |  |  |  |  |
|  |  | 2 🡪 > 30% |  |  |  |  |  |  |  |  |  |  |
|  | Percentage of voluntary savers compared to total recipients of the fund | 0 🡪 < 50% | 228 | 2 | 147 | 2 | 125 | 2 | 120 | 2 | 104 | 2 |
|  |  | 1 🡪 50% - 75% |  |  |  |  |  |  |  |  |  |  |
|  |  | 2 🡪 > 75% |  |  |  |  |  |  |  |  |  |  |
|  | Percentage of members who that use MFI services such as transfers or insurance to the total recipient of the fund | 0 🡪 < 10% | 20 | 1 | 20 | 1 | 60 | 2 | 60 | 2 | 80 | 2 |
|  |  | 1 🡪 10% - 30% |  |  |  |  |  |  |  |  |  |  |
|  |  | 2 🡪 > 30% |  |  |  |  |  |  |  |  |  |  |
|  | Percentage of members that received non-financial facilities such as training or other empowerment programs to the total recipient of the fund | 0 🡪 < 10% | 0 | 0 | 0 | 0 | 7 | 0 | 0 | 0 | 7 | 0 |
|  |  | 1 🡪 10% - 30% |  |  |  |  |  |  |  |  |  |  |
|  |  | 2 🡪 > 30% |  |  |  |  |  |  |  |  |  |  |
| Total | | |  | 3 |  | 3 |  | 5 |  | 5 |  | 5 |
| Breadth Outreach Score (Total Score : 10) | | |  | 0.3 |  | 0.3 |  | 0.5 |  | 0.5 |  | 0.5 |
| Depth | The average percentage of funding provided to members per Gross Domestic Regional Product per capita in East Java | 0 🡪 > 100% | 99 | 1 | 50 | 2 | 45 | 2 | 45 | 2 | 54 | 2 |
|  |  | 1 🡪 60% - 100% |  |  |  |  |  |  |  |  |  |  |
|  |  | 2 🡪 < 60% |  |  |  |  |  |  |  |  |  |  |
|  | Percentage of financing under $ 300 (equivalent to Rp.4,027,500) | 0 🡪 < 20% | 10 | 0 | 10 | 0 | 5 | 0 | 6 | 0 | 6 | 0 |
|  |  | 1 🡪 20% - 50% |  |  |  |  |  |  |  |  |  |  |
|  |  | 2 🡪 > 50% |  |  |  |  |  |  |  |  |  |  |
|  | Percentage of number of female financing recipients | 0 🡪 < 20% | 9 | 0 | 10 | 0 | 10 | 0 | 13 | 0 | 11 | 0 |
|  |  | 1 🡪 20% - 50% |  |  |  |  |  |  |  |  |  |  |
|  |  | 2 🡪 > 50% |  |  |  |  |  |  |  |  |  |  |
|  | Percentage of recipients of funding from villages | 0 🡪 < 15% | 70 | 2 | 73 | 2 | 80 | 2 | 80 | 2 | 80 | 2 |
|  |  | 1 🡪 15% - 30% |  |  |  |  |  |  |  |  |  |  |
|  |  | 2 🡪 > 30% |  |  |  |  |  |  |  |  |  |  |
|  | Percentage of financing to entrepreneurs originating from poor customers targeted by the poverty alleviation programs | 0 🡪 < 20% | 7 | 0 | 7 | 0 | 6 | 0 | 5 | 0 | 3 | 0 |
|  |  | 1 🡪 20% - 50% |  |  |  |  |  |  |  |  |  |  |
|  |  | 2 🡪 > 50% |  |  |  |  |  |  |  |  |  |  |
| Total | | |  | 3 |  | 4 |  | 4 |  | 4 |  | 4 |
| Depth Outreach Score (Total Score : 10) | | |  | 0.3 |  | 0.4 |  | 0.4 |  | 0.4 |  | 0.4 |
| Total of Outreach Score (Total of Breadth and Depth Score) | | |  | 6 |  | 7 |  | 9 |  | 9 |  | 9 |
| Overall Outreach Score (Total of Outreach Score : 20) | | |  | 0.3 |  | 0.35 |  | 0.45 |  | 0.45 |  | 0.45 |

| **Mandiri Artha Syariah** | |  |  |  |  |  |  |  |  |  |  |  |
| --- | --- | --- | --- | --- | --- | --- | --- | --- | --- | --- | --- | --- |
| Outreach | Indicator | Scale | 2014 | | 2015 | | 2016 | | 2017 | | 2018 | |
|  |  |  | Data | Score | Data | Score | Data | Score | Data | Score | Data | Score |
| Breadth | The Number of Financing Receiver | 0 🡪 < 20.000 | 15 | 0 | 100 | 0 | 400 | 0 | 1800 | 0 | 2500 | 0 |
|  |  | 1 🡪 20.000 – 50.000 |  |  |  |  |  |  |  |  |  |  |
|  |  | 2 🡪 > 50.000 |  |  |  |  |  |  |  |  |  |  |
|  | The percentage of financial aid recipients used for non-productive activities | 0 🡪 < 10% | 30 | 1 | 20 | 1 | 20 | 1 | 15 | 1 | 10 | 1 |
|  |  | 1 🡪 10% - 30% |  |  |  |  |  |  |  |  |  |  |
|  |  | 2 🡪 > 30% |  |  |  |  |  |  |  |  |  |  |
|  | Percentage of voluntary savers compared to total recipients of the fund | 0 🡪 < 50% | 333 | 2 | 200 | 2 | 1000 | 2 | 322 | 2 | 240 | 2 |
|  |  | 1 🡪 50% - 75% |  |  |  |  |  |  |  |  |  |  |
|  |  | 2 🡪 > 75% |  |  |  |  |  |  |  |  |  |  |
|  | Percentage of members who that use MFI services such as transfers or insurance to the total recipient of the fund | 0 🡪 < 10% | 0 | 0 | 5 | 0 | 10 | 1 | 60 | 2 | 70 | 2 |
|  |  | 1 🡪 10% - 30% |  |  |  |  |  |  |  |  |  |  |
|  |  | 2 🡪 > 30% |  |  |  |  |  |  |  |  |  |  |
|  | Percentage of members that received non-financial facilities such as training or other empowerment programs to the total recipient of the fund | 0 🡪 < 10% | 1 | 0 | 4 | 0 | 10 | 1 | 15 | 1 | 50 | 2 |
|  |  | 1 🡪 10% - 30% |  |  |  |  |  |  |  |  |  |  |
|  |  | 2 🡪 > 30% |  |  |  |  |  |  |  |  |  |  |
| Total | | |  | 3 |  | 3 |  | 5 |  | 6 |  | 7 |
| Breadth Outreach Score (Total Score : 10) | | |  | 0.3 |  | 0.3 |  | 0.5 |  | 0.6 |  | 0.7 |
| Depth | The average percentage of funding provided to members per Gross Domestic Regional Product per capita in East Java | 0 🡪 > 100% | 201 | 0 | 28 | 2 | 7 | 2 | 1 | 2 | 1 | 2 |
|  |  | 1 🡪 60% - 100% |  |  |  |  |  |  |  |  |  |  |
|  |  | 2 🡪 < 60% |  |  |  |  |  |  |  |  |  |  |
|  | Percentage of financing under $ 300 (equivalent to Rp.4,027,500) | 0 🡪 < 20% | 90 | 2 | 85 | 2 | 70 | 2 | 60 | 2 | 40 | 1 |
|  |  | 1 🡪 20% - 50% |  |  |  |  |  |  |  |  |  |  |
|  |  | 2 🡪 > 50% |  |  |  |  |  |  |  |  |  |  |
|  | Percentage of number of female financing recipients | 0 🡪 < 20% | 90 | 2 | 80 | 2 | 70 | 2 | 70 | 2 | 73 | 2 |
|  |  | 1 🡪 20% - 50% |  |  |  |  |  |  |  |  |  |  |
|  |  | 2 🡪 > 50% |  |  |  |  |  |  |  |  |  |  |
|  | Percentage of recipients of funding from villages | 0 🡪 < 15% | 100 | 2 | 100 | 2 | 100 | 2 | 100 | 2 | 100 | 2 |
|  |  | 1 🡪 15% - 30% |  |  |  |  |  |  |  |  |  |  |
|  |  | 2 🡪 > 30% |  |  |  |  |  |  |  |  |  |  |
|  | Percentage of financing to entrepreneurs originating from poor customers targeted by the poverty alleviation programs | 0 🡪 < 20% | 100 | 2 | 80 | 2 | 70 | 2 | 75 | 2 | 60 | 2 |
|  |  | 1 🡪 20% - 50% |  |  |  |  |  |  |  |  |  |  |
|  |  | 2 🡪 > 50% |  |  |  |  |  |  |  |  |  |  |
| Total | | |  | 8 |  | 10 |  | 10 |  | 10 |  | 9 |
| Depth Outreach Score (Total Score : 10) | | |  | 0.8 |  | 1 |  | 1 |  | 1 |  | 0.9 |
| Total of Outreach Score (Total of Breadth and Depth Score) | | |  | 11 |  | 13 |  | 15 |  | 16 |  | 16 |
| Overall Outreach Score (Total of Outreach Score : 20) | | |  | 0.55 |  | 0.65 |  | 0.75 |  | 0.8 |  | 0.8 |

| **Mandiri Artha Syariah** | |  |  |  |  |  |  |  |  |  |  |  |
| --- | --- | --- | --- | --- | --- | --- | --- | --- | --- | --- | --- | --- |
| Outreach | Indicator | Scale | 2014 | | 2015 | | 2016 | | 2017 | | 2018 | |
|  |  |  | Data | Score | Data | Score | Data | Score | Data | Score | Data | Score |
| Breadth | The Number of Financing Receiver | 0 🡪 < 20.000 | 545 | 0 | 495 | 0 | 412 | 0 | 335 | 0 | 296 | 0 |
|  |  | 1 🡪 20.000 – 50.000 |  |  |  |  |  |  |  |  |  |  |
|  |  | 2 🡪 > 50.000 |  |  |  |  |  |  |  |  |  |  |
|  | The percentage of financial aid recipients used for non-productive activities | 0 🡪 < 10% | 15 | 1 | 15 | 1 | 20 | 1 | 10 | 1 | 25 | 1 |
|  |  | 1 🡪 10% - 30% |  |  |  |  |  |  |  |  |  |  |
|  |  | 2 🡪 > 30% |  |  |  |  |  |  |  |  |  |  |
|  | Percentage of voluntary savers compared to total recipients of the fund | 0 🡪 < 50% | 136 | 2 | 131 | 2 | 176 | 2 | 221 | 2 | 227 | 2 |
|  |  | 1 🡪 50% - 75% |  |  |  |  |  |  |  |  |  |  |
|  |  | 2 🡪 > 75% |  |  |  |  |  |  |  |  |  |  |
|  | Percentage of members who that use MFI services such as transfers or insurance to the total recipient of the fund | 0 🡪 < 10% | 80 | 2 | 85 | 2 | 85 | 2 | 85 | 2 | 90 | 2 |
|  |  | 1 🡪 10% - 30% |  |  |  |  |  |  |  |  |  |  |
|  |  | 2 🡪 > 30% |  |  |  |  |  |  |  |  |  |  |
|  | Percentage of members that received non-financial facilities such as training or other empowerment programs to the total recipient of the fund | 0 🡪 < 10% | 5 | 0 | 4 | 0 | 5 | 0 | 4 | 0 | 5 | 0 |
|  |  | 1 🡪 10% - 30% |  |  |  |  |  |  |  |  |  |  |
|  |  | 2 🡪 > 30% |  |  |  |  |  |  |  |  |  |  |
| Total | | |  | 5 |  | 5 |  | 5 |  | 5 |  | 5 |
| Breadth Outreach Score (Total Score : 10) | | |  | 0.5 |  | 0.5 |  | 0.5 |  | 0.5 |  | 0.5 |
| Depth | The average percentage of funding provided to members per Gross Domestic Regional Product per capita in East Java | 0 🡪 > 100% | 22 | 2 | 20 | 2 | 21 | 2 | 21 | 2 | 23 | 2 |
|  |  | 1 🡪 60% - 100% |  |  |  |  |  |  |  |  |  |  |
|  |  | 2 🡪 < 60% |  |  |  |  |  |  |  |  |  |  |
|  | Percentage of financing under $ 300 (equivalent to Rp.4,027,500) | 0 🡪 < 20% | 20 | 1 | 20 | 1 | 25 | 1 | 15 | 0 | 10 | 0 |
|  |  | 1 🡪 20% - 50% |  |  |  |  |  |  |  |  |  |  |
|  |  | 2 🡪 > 50% |  |  |  |  |  |  |  |  |  |  |
|  | Percentage of number of female financing recipients | 0 🡪 < 20% | 75 | 2 | 75 | 2 | 75 | 2 | 80 | 2 | 70 | 2 |
|  |  | 1 🡪 20% - 50% |  |  |  |  |  |  |  |  |  |  |
|  |  | 2 🡪 > 50% |  |  |  |  |  |  |  |  |  |  |
|  | Percentage of recipients of funding from villages | 0 🡪 < 15% | 6 | 0 | 8 | 0 | 5 | 0 | 5 | 0 | 5 | 0 |
|  |  | 1 🡪 15% - 30% |  |  |  |  |  |  |  |  |  |  |
|  |  | 2 🡪 > 30% |  |  |  |  |  |  |  |  |  |  |
|  | Percentage of financing to entrepreneurs originating from poor customers targeted by the poverty alleviation programs | 0 🡪 < 20% | 15 | 0 | 10 | 0 | 10 | 0 | 10 | 0 | 5 | 0 |
|  |  | 1 🡪 20% - 50% |  |  |  |  |  |  |  |  |  |  |
|  |  | 2 🡪 > 50% |  |  |  |  |  |  |  |  |  |  |
| Total | | |  | 5 |  | 5 |  | 5 |  | 4 |  | 4 |
| Depth Outreach Score (Total Score : 10) | | |  | 0.5 |  | 0.5 |  | 0.5 |  | 0.4 |  | 0.4 |
| Total of Outreach Score (Total of Breadth and Depth Score) | | |  | 10 |  | 10 |  | 10 |  | 9 |  | 9 |
| Overall Outreach Score (Total of Outreach Score : 20) | | |  | 0.5 |  | 0.5 |  | 0.5 |  | 0.45 |  | 0.45 |
